# Multiplex PCR and Antibiotic Use in Children with Community-Acquired Pneumonia

**DOI:** 10.3390/children11020245

**Published:** 2024-02-15

**Authors:** Teresa del Rosal, Patricia Bote-Gascón, Iker Falces-Romero, Talía Sainz, Fernando Baquero-Artigao, Paula Rodríguez-Molino, Ana Méndez-Echevarría, Blanca Bravo-Queipo-de-Llano, Luis A. Alonso, Cristina Calvo

**Affiliations:** 1 Pediatrics and Infectious Diseases Department, Institute for Health Research IdiPAZ, Hospital Universitario La Paz, Paseo de la Castellana 261, 28046 Madrid, Spain; patricia.bote@salud.madrid.org (P.B.-G.); talia.sainz@salud.madrid.org (T.S.); fernando.baquero@salud.madrid.org (F.B.-A.); prmolino@salud.madrid.org (P.R.-M.); ana.mendez@salud.madrid.org (A.M.-E.); luisalfonso.alonso@salud.madrid.org (L.A.A.); ccalvor@salud.madrid.org (C.C.); 2 Translational Research Network in Pediatric Infectious Diseases (RITIP), 28046 Madrid, Spain; 3 Center for Biomedical Network Research on Rare Diseases (CIBERER U767, Instituto de Salud Carlos III), 28029 Madrid, Spain; 4 Pediatric Emergency Department, Hospital Universitario La Paz, 28046 Madrid, Spain; 5 Microbiology Department, Hospital Universitario La Paz, 28046 Madrid, Spain; falces88@gmail.com; 6 Center for Biomedical Network Research on Infectious Diseases (CIBERINFEC, Instituto de Salud Carlos III), 28029 Madrid, Spain; 7 Department of Pediatrics, Faculty of Medicine, Universidad Autónoma de Madrid, 28029 Madrid, Spain

**Keywords:** pneumonia, multiplex polymerase chain reaction, viruses, anti-bacterial agents

## Abstract

Antibiotics are frequently prescribed to children with pneumonia, although viruses are responsible for most cases. We aimed to evaluate the impact of multiplex polymerase chain reaction (mPCR) on antibiotic use. We conducted a prospective study of children under 14 years of age admitted for suspected viral pneumonia, from October 2019 to June 2022 (except March–November 2020). A mPCR respiratory panel (FilmArray^®^ 2*plus*, bioMérieux, Marcy-l’Étoile, France) was performed within 72 h of admission. Patients with positive reverse transcription PCR for respiratory syncytial virus, influenza, or SARS-CoV-2 were excluded. We compared the patients with historical controls (2017–2018) who had suspected viral pneumonia but did not undergo an aetiological study. We included 64 patients and 50 controls, with a median age of 26 months. The respiratory panel detected viral pathogens in 55 patients (88%), including 17 (31%) with co-infections. Rhinovirus/enterovirus (*n* = 26) and human metapneumovirus (*n* = 22) were the most common pathogens, followed by adenovirus and parainfluenza (*n* = 10). There were no statistically significant differences in the total antibiotic consumption (83% of cases and 86% of controls) or antibiotics given for ≥72 h (58% vs. 66%). Antibiotics were prescribed in 41% of the cases and 72% of the controls at discharge (*p* = 0.001). Ampicillin was the most commonly prescribed antibiotic among the patients (44% vs. 18% for controls, *p* = 0.004), while azithromycin was the most commonly prescribed among the controls (19% vs. 48% for patients and controls, respectively; *p* = 0.001). Our findings underscore the need for additional interventions alongside molecular diagnosis to reduce antibiotic usage in paediatric community-acquired pneumonia.

## 1. Introduction

Community-acquired pneumonia (CAP) is a leading cause of childhood morbidity and mortality worldwide. Due to this large disease burden on paediatric health, the optimal management of CAP is essential, including prompt diagnosis and appropriate antimicrobial therapy. In recent years, the wide use of molecular diagnostic tests for identifying the aetiology of respiratory infections has underscored the significant contribution of respiratory viruses to paediatric CAP. However, although respiratory viruses are responsible for most cases of CAP, antibiotics are often prescribed [1]. 

Bacterial diagnostics remain suboptimal [1], and the distinction between viral and bacterial CAP is usually based on the combined evaluation of epidemiological factors (patient’s age, viral epidemics in the community), clinical and radiological manifestations, and inflammatory markers. However, there is significant overlap, and clinical algorithms cannot clearly discern the cause of CAP [2], which leads to substantial variations in its management. Various diagnostic models have been developed to predict the viral aetiology of paediatric respiratory infections, but most have focused on specific viruses, mainly influenza, and are not yet ready for clinical application [3]. Moreover, the identification of viruses within the upper respiratory tract does not confirm their causal relationship with CAP, nor does it exclude the presence of a bacterial pathogen, given the frequent occurrence of mixed viral–bacterial infections in paediatric cases [2,4]. The detection of certain respiratory viruses (respiratory syncytial virus, metapneumovirus, influenza and parainfluenza) is likely causative of the disease, whereas the clinical significance of other respiratory pathogens is less clear [5,6].

In this context, the detection of a plausible viral agent might lead to a significant reduction in antibiotic prescriptions, decreasing the use of unnecessary antibiotics, the occurrence of adverse drug reactions and the emergence of drug-resistant strains [7]. Prior studies that have included children with respiratory infections have shown how point-of-care microbiological tests are associated with a decrease in antibiotic use. However, results have been inconsistent across various series. The fact that the inclusion criteria show ample variation [8] might underline some of the differences encountered among the studies performed using multiplex polymerase chain reaction (mPCR) in paediatric hospitalised patients, given that most studies did not restrict the study population to a specific diagnosis such as CAP [9,10,11,12]. Therefore, the impact of mPCR on antibiotic prescription in children with suspected viral CAP remains unclear. Various factors can further influence antibiotic utilization, such as apprehension stemming from the patient’s characteristics or the physician’s uncertainty, inadequate knowledge, and a sense of complacency [13,14].

We designed this study to evaluate the effect of mPCR on antibiotic consumption in paediatric inpatients with suspected viral CAP compared to the standard clinical practice.

## 2. Patients and Methods

### 2.1. Study Design

This was a prospective study of children diagnosed with suspected viral CAP and admitted to a tertiary paediatric hospital (La Paz University Hospital, Madrid, Spain), and recruited from October 2019 to June 2022. The study was interrupted between 12 March and 9 November 2020 due to the COVID-19 pandemic. A mPCR respiratory panel was performed in the first 72 h after hospital admission. Patients were compared with a historic cohort of children admitted for suspected viral CAP during the previous seasons (January 2017–April 2019) when mPCR was not available. The control group was identified using discharge codes from the International Classification of Diseases, Tenth Revision (ICD-10), specifically J.12 for viral pneumonia, J.16 for pneumonia due to other infectious organisms not elsewhere classified, and J18 for pneumonia of unspecified organism. Subsequently, the control group’s electronic medical records underwent a thorough review to ensure that selected cases met the diagnostic criteria consistent with suspected viral CAP, mirroring those applied to the patient group, and did not meet any exclusion criteria. Lastly, the patients and controls were matched by age (in years) and corresponding seasons to enhance comparability between the groups.

Data were collected anonymously and included clinical and epidemiological characteristics (age, sex, underlying health conditions, signs and symptoms) and laboratory, radiology and microbiology results. We also recorded data regarding antibiotic prescription (total antibiotic use during hospital say, antibiotic use for more than 72 h, antibiotic prescription at discharge, and duration of antibiotic therapy), length of hospital stay and paediatric intensive care unit admission. 

The study was approved by the local ethics committee (study number PI-3703), and written informed consent was obtained from the parents or legal guardians before study inclusion.

### 2.2. Definitions and Inclusion/Exclusion Criteria

The parents of patients under 16 years of age admitted to La Paz University Hospital with a diagnosis of suspected viral CAP were offered the opportunity of enrolling their children in the study. 

CAP was defined as an acute lower respiratory tract infection that had started in the past 14 days, with fever (body temperature > 37.8 °C) and respiratory symptoms (at least one of the following: cough, sputum production, pleuritic pain, poor appetite), and/or abnormal physical examination (at least one of the following: tachypnoea, breathing difficulty, abnormal lung auscultation), and new or increasing alveolar infiltrate in chest radiography. In children younger than 2 years, peribronchial thickening and diffuse small patchy infiltrates were not considered as alveolar infiltrates unless there was also pleural effusion.

The type of CAP was established according to the following criteria [15]:

- Acute onset of fever ≥ 39 °C
- Pleuritic chest pain or equivalent (abdominal pain, meningismus)
- Focal lung auscultation (tubal murmur, hypoventilation or crackles)
- Focal consolidation in chest radiographs
- Leucocytosis > 12,000/mm^3^ with neutrophilia > 6000/mm^3^
- C-reactive protein (CRP) level > 60 mg/L

Patients fulfilling fewer than 3 criteria were classified as having suspected viral CAP, whereas those with 3 or more criteria were considered as having typical bacterial CAP. 

Children were excluded if they were immunocompromised or had been discharged from hospital in the previous 3 days before the current admission. We also excluded those patients with positive PCR for influenza or respiratory syncytial virus (RSV), which is routinely performed at our institution prior to hospital admission on all children with respiratory infections (Cobas^®^ Liat Influenza A/B & RSV, Roche Diagnostics, Basel, Switzerland). From November 2020, we also excluded children who tested positive for SARS-CoV-2, which was tested through several commercial RT-PCR assays and/or rapid antigen tests on nasopharyngeal swabs (including TaqMan 2019 nCoV Assay Kit v1 [Thermo Fisher Scientific Inc., Franklin, MO, USA], SARS-CoV-2 Real Time PCR Kit, [Vircell, Granada, Spain] and Seegene Allplex SARS-CoV-2 Assay [Seegene, Seoul, Republic of Korea]).

### 2.3. Microbiological Analysis

Nasopharyngeal swabs were collected in the first 72 h after hospital admission by paediatric nurses according to the standard procedure. The swabs were immediately sent to the Microbiology Department, where they were processed and tested using the FilmArray^®^ Respiratory 2*plus* Panel (bioMérieux, Marcy-l’Étoile, France), which has high sensitivity and specificity (97.1% and 99.3%, according to the manufacturer’s data [16]) and includes the following viruses and bacteria: adenovirus, coronavirus 229E, coronavirus HKU1, coronavirus OC43, coronavirus NL63, Middle East respiratory syndrome coronavirus, human metapneumovirus (hMPV), human rhinovirus/enterovirus (RV/EV), influenza A, influenza A/H1, influenza A/H1-2009, influenza A/H3, influenza B, parainfluenza 1, parainfluenza 2, parainfluenza 3, parainfluenza 4, RSV, *Bordetella pertussis*, *Bordetella parapertussis*, *Chlamydophila pneumoniae* and *Mycoplasma pneumoniae*.

The results were reported to the attending physician on the same day the swabs were collected. The interpretation of the PCR results was primarily guided by the treating paediatrician’s usual practice and clinical judgement.

### 2.4. Statistical Analysis

Quantitative variables are reported as the median and interquartile range (IQR), and qualitative variables are listed as absolute and relative frequencies. We compared the patients and controls and compared the patients according to the positive/negative pathogen identification and probable causative agent (hMPV, influenza, parainfluenza, RSV, *Bordetella pertussis*, *Bordetella parapertussis*, *Chlamydophila pneumoniae* and *Mycoplasma pneumoniae*) or non-causative agent (RV/EV, coronaviruses, adenovirus). We also compared the children who were administered antibiotics with those who were not. The quantitative variables were compared using the Mann–Whitney U test, while the qualitative variables were compared using Pearson’s chi-squared or Fisher’s exact test. A logistic regression analysis was employed to examine the association between antibiotic prescription and laboratory parameters. We considered as statistically significant those results with a *p*-value < 0.05. Given the minimal amount of missing data in the dataset, no specific methods for handling missing data were employed. The data were analysed using the Statistical Package for the Social Sciences (SPSS) software (SPSS for Windows, version 25.0, IBM SPSS Corp.; Armonk, NY, USA).

## 3. Results

### 3.1. Characteristics of the Patients and Controls

We included 64 patients and 50 matched controls with a median age of 26 months, whose main characteristics are listed in Table 1. Blood cultures were performed for 49 patients and 28 controls, all of which were negative or contaminated (one in the patient group and three in the control group). The patients had higher CRP levels, more frequently showed consolidations in the chest X-rays and had longer hospital stays, despite being matched by age and season.

### 3.2. Multiplex PCR Results

**Figure 1:**
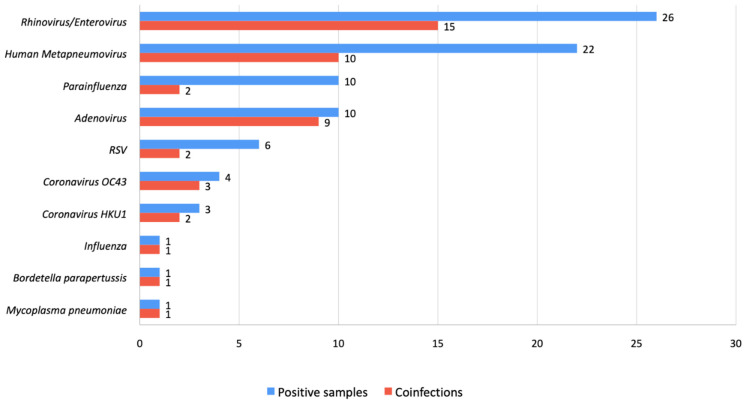
Microbiological results of the children with positive mPCR (*n* = 55). Abbreviations: RSV, respiratory syncytial virus.

The FilmArray^®^ Respiratory 2*plus* panel was positive in 55 patients (86%) (Figure 1). In 17 cases (31% of those with positive mPCR), the test detected more than one pathogen: seven cases with two pathogens, eight cases with three, and two cases with four. The most commonly identified pathogens were RV/EV (*n* = 26) and hMPV (*n* = 22), followed by adenovirus (*n* = 10), parainfluenza (*n* = 10), RSV (*n* = 6), coronavirus OC43 (*n* = 4) and coronavirus HKU1 (*n* = 3). There was also one positive case each of influenza A, *Bordetella parapertussis* and *Mycoplasma pneumoniae*.

The patients with RSV or influenza had tested negative for those viruses before hospital admission. The most frequent coinfections were RV/EV and hMPV (in eight cases) and adenovirus and hMPV (in five cases). In 21 cases (38% of those with positive viral detection), the only pathogens identified were RV/EV, coronavirus OC43 or HKU1 or adenovirus. 

### 3.3. Antibiotic Prescription

Antibiotics were prescribed to more than 80% of the children in both the patient and control groups, with a median treatment duration of 7 days (IQR 2–8.5) for the patient group and 6 days (IQR 3–8) for the control group (*p* = 0.764) (Table 2). At hospital discharge, antibiotics were prescribed to 41% of the patients and 72% of the controls (*p* = 0.001). Ampicillin was the most commonly prescribed antibiotic among the patients (44% vs. 18% of the controls, *p* = 0.005), whereas azithromycin was the most common among the control group (19% of the patients vs. 48% of the controls, *p* = 0.001).

When restricting the analysis to those patients in whom mPCR was performed, we found no positive effect for the antibiotic use. Forty-six patients (84%) with at least one pathogen detected in the respiratory panel were administered antibiotics, compared with seven patients (78%) with negative results (*p* = 0.646). There were also no statistically significant differences in antibiotic prescription for ≥72 h (55% for those with positive results vs. 78%, *p* = 0.282), antibiotics at discharge (36% vs. 67%, *p* = 0.142) and the duration of antibiotic therapy (median 6 days [IQR 2–8] vs. 8 days [IQR 5–10], *p* = 0.129). There were also no differences when comparing the patients with the isolation of RV/EV, coronavirus or adenovirus with those with other pathogens (Table 3).

We analysed the effect of various factors on antibiotic prescription in the whole cohort (patients and controls). In the bivariate analysis, the children with higher CRP levels, leukocyte counts and neutrophil counts were more frequently prescribed antibiotics, whereas there was no association between age or paediatric intensive care unit admission and antibiotic use (Table 4). 

A logistic regression was performed to ascertain the effects of the laboratory parameters on the likelihood that the children were prescribed antibiotics. Only increased CRP levels were associated with a higher likelihood of antibiotic prescription (*p* = 0.041). 

## 4. Discussion

In this series of children admitted for suspected viral CAP, we detected at least one respiratory virus in almost 90% of the children and coinfections in a third of them. Our hypothesis was that the availability of mPCR would have an impact on antibiotic prescription, given that it has been previously reported in children with acute respiratory infections [8]. However, although mPCR testing resulted in decreased antibiotic prescription at discharge, it did not lead to a decrease in overall antibiotic use during hospitalization in our study. Several factors might have contributed to this finding.

Prior studies conducted with paediatric inpatients have included children with various acute respiratory infections, such as bronchiolitis [9,10,11,12,17], whereas we employed a stringent definition of CAP. In bronchiolitis, several evidence-based guidelines that emphasize the avoidance of unnecessary treatments and investigations have been published, leading to decreased antibiotic use among paediatric inpatients [18]. On the other hand, the use of antibiotics in paediatric CAP is still higher than expected, and viral infections probably account for a large proportion of prescriptions due to the limited accuracy of biomarkers to distinguish between bacterial and viral infections and the limitations of current microbiological tests to identify bacteria [1]. 

The impact of mPCR on children with lower respiratory tract infections might vary in different settings. Studies conducted among ward and paediatric critical care patients have shown a modest effect on antimicrobial prescriptions [19,20], whereas among emergency department patients, mPCR is associated with a decrease in diagnostic investigations and antibiotic prescriptions [21]. A number of studies have shown that the test turnaround time could be an important factor influencing antibiotic prescription [22,23]. Although the results were available on the same day the swabs were collected in our study, we allowed for mPCR to be performed in the first 72 h after hospital admission, and most patients were already taking antibiotics. A prospective cohort study of children hospitalized with suspected CAP showed that antibiotics initiated in the emergency department were continued in the inpatient setting in 90% of the children [24]. Therefore, for children who are going to be hospitalized, mPCR testing could be optimized by using it as a point-of-care test, waiting for its results before initiating antibiotic therapy. These patients are periodically reassessed; in case of worsening and suspected mixed viral–bacterial infection, antibiotic therapy can be promptly introduced.

Viral detection by molecular testing in an upper respiratory tract sample does not rule out co-infection by bacteria [25]. Moreover, certain respiratory viruses (RV/EV, human coronaviruses, adenovirus) have frequently been detected in asymptomatic children, and the identification of these viruses in patients with CAP should be interpreted with caution [5,6]. In our series, the patients with infections caused by these viruses with no other co-infection accounted for more than one third of those with positive viral detections. In these cases, clinicians might be more reluctant to stop the antibiotic therapy. However, hMPV and parainfluenza viruses are likely to be the cause of the disease in children with acute respiratory infections [5] and accounted for more than half of the positive results in our series. We experienced an unusual outbreak of hMPV infections in the fall of 2021, with higher rates of pneumonia and antibiotic therapy than for those admitted during the 2005–2020 period [26]. In our series, hMPV was the second most common pathogen, and the C-reactive protein levels were higher among the children who underwent mPCR testing than those among the historical controls. Prior studies among paediatric inpatients have shown that patients with hMPV infections are more likely to be administered antibiotics than patients with other respiratory viruses [10,27].

CRP is one of the best biomarkers to differentiate between bacterial and viral pneumonia. A recent meta-analysis determined that 53 mg/L is the statistically optimal cut-off based on the Youden index, but its sensitivity and specificity were suboptimal (approximately 70% and 65%, respectively) [25]. In our cohort, the higher CRP results were associated with a higher likelihood of antibiotic prescription, probably because clinicians felt that they could not rule out a bacterial coinfection.

Our study highlights the need for antimicrobial stewardship strategies for children with CAP, as well as diagnostic tests to improve antibiotic prescription. It is important to have guidelines and tools that help clinicians distinguish viral from bacterial pneumonia [3] and to make appropriate treatment decisions, thereby helping to reduce unnecessary antibiotic therapy for viral infections. Active antimicrobial stewardship could be another important approach. Prior studies have shown a higher likelihood of antibiotic prescription reduction in settings with active antimicrobial stewardship, a low likelihood of bacterial infection, and short turnaround times. The use of mPCR as an isolated intervention improves microbiological diagnosis but does not consistently decrease antimicrobial utilization [28,29]. 

Although this study provides valuable insights, it has several limitations. The study was conducted at a referral children’s hospital, and the findings might not be applicable to other populations. The benefits of mPCR might vary in different settings according to the standard clinical practices, physician attitudes, microbiological test availability, and characteristics of the healthcare systems, among other factors. We were unable to analyse the differences between pathogens owing to the small sample size. Our comparison with historical controls lacked perfect alignment in terms of patient characteristics. Shifts in population characteristics, viral pathogen circulation and healthcare utilization patterns over time might also contribute to the variability in antibiotic-prescribing practices and CAP management strategies. However, there were no major changes in the diagnostic techniques and hospital protocols used during the study period, except during the COVID-19 pandemic, which caused significant changes in respiratory virus infections worldwide [30].

## 5. Conclusions

Our study showed no decrease in antibiotic prescription during the hospital stay of children with suspected viral CAP. Several factors might have contributed to this finding, including the high prevalence of hMPV infection and the need for better antimicrobial stewardship strategies. Future studies should focus on methods able to optimize the use of mPCR testing.

## Figures and Tables

**Table 1:** Characteristics of the patients and controls with suspected viral community-acquired pneumonia. Data are median (interquartile range) unless otherwise indicated. Abbreviations: CRP, C-reactive protein; ICU, intensive care unit. ^a^ Defined as abnormal paediatric assessment triangle. ^b^ Defined as peripheral arterial oxygen saturation ≤ 92%.

	Patients (*n* = 64)	Controls (*n* = 50)	*p*
**Age, months**	26 (18–43)	26 (15–50.5)	0.895
**Female sex, *n* (%)**	36 (56%)	24 (48%)	0.381
**Prematurity, *n* (%)**	12 (19%)	5 (10%)	0.193
**Underlying medical conditions, *n* (%)**	14 (22%)	10 (20%)	0.807
**Clinical characteristics**			
Breathing difficulty, *n* (%)	62 (97%)	45 (90%)	0.237
Wheezing, *n* (%)	51 (80%)	38 (76%)	0.655
Appears unwell ^a^, *n* (%)	12 (19%)	4 (8%)	0.113
Hypoxaemia ^b^, *n* (%)	59 (92%)	42 (84%)	0.237
**Laboratory results**			
Leukocyte count/mm^3^	10,310 (7700–13,828)	11,800 (8000–15,850)	0.642
Neutrophil count/mm^3^	6815 (4565–9858)	7397 (3603–11,755)	0.947
CRP (mg/L)	28.7 (13.5–72)	12.5 (4.7–23.6)	<0.0001
**Chest X-ray results**			
Infiltrates, *n* (%)	31 (48%)	35 (70%)	0.002
Focal consolidation, *n* (%)	32 (50%)	14 (28%)	0.0175
Pleural effusion, *n* (%)	1 (1.6%)	1 (2%)	1
**ICU admission, *n* (%)**	7 (11%)	5 (10%)	1
**Hospital stay, days**	4 (3–6)	3 (2–4.3)	<0.0001

**Table 2:** Antibiotic prescription for the patients and controls with suspected viral community-acquired pneumonia. Data are *n* (%) unless otherwise indicated.

	Patients (*n* = 64)	Controls (*n* = 50)	*p*
**Any antibiotic treatment**	53 (83%)	43 (86%)	0.643
**Antibiotics ≥ 72 h**	37 (58%)	33 (66%)	0.373
**Antibiotics after discharge**	26 (41%)	36 (72%)	0.001
**Total antibiotic duration, days; median (IQR)**	7 (2–8.5)	6 (3–8)	0.764
**Antibiotics**			
Ampicillin	28 (44%)	9 (18%)	0.004
Amoxicillin	20 (31%)	14 (28%)	0.707
Amoxicillin/clavulanate	13 (20%)	13 (26%)	0.473
Azithromycin	12 (19%)	24 (48%)	0.001
Cefotaxime or ceftriaxone	6 (9%)	1 (2%)	0.421

**Table 3:** Antibiotic prescription for the patients with suspected viral community-acquired pneumonia, according to identified pathogens. Data are presented as median (interquartile range) unless otherwise indicated. Abbreviations: ADV, adenovirus; CoV, coronavirus OC43 and HKU1, RV/EV, human rhinovirus/enterovirus.

	RV/EV, CoV, ADV (*n* = 21)	Other Pathogens (*n* = 34)	*p*
Any antibiotic treatment, *n* (%)	18 (86%)	28 (82%)	1
Antibiotics ≥ 72 h, *n* (%)	10 (48%)	20 (59%)	0.578
Antibiotics after discharge, *n* (%)	6 (29%)	14 (41%)	0.399
Total antibiotic duration	5.5 (1–8.25)	7 (2–8)	0.459
Hospital stay, days	5 (4–6.5)	4 (2–7)	0.372

**Table 4:** Comparison of age, laboratory parameters and ICU admission according to antibiotic prescription. Data are median (interquartile range) unless otherwise indicated. Abbreviations: CRP, C-reactive protein; ICU, intensive care unit.

	Antibiotics (*n* = 96)	No antibiotics (*n* = 18)	*p*
Age, months	26.5 (17.3–48.8)	22 (9.5–42.3)	0.104
Leukocyte count/mm^3^	11,730 (7850–15,910)	9515 (7072–10,730)	0.034
Neutrophil count/mm^3^	7425 (4515–11,755)	5920 (4296–7602)	0.067
CRP (mg/L)	23.9 (10.5–50.6)	12.3 (6.1–18.9)	0.005
ICU admission, *n* (%)	11 (12%)	1 (6%)	0.688

## Data Availability

The data presented in this study are available on request from the corresponding author (containing information that could compromise the privacy of research participants).
